# Possible therapeutic targets for SARS-CoV-2 infection and COVID-19

**DOI:** 10.46439/allergy.2.028

**Published:** 2021

**Authors:** Nabab Khan, Nirmal Kumar, Jonathan D. Geiger

**Affiliations:** Department of Biomedical Sciences, University of North Dakota School of Medicine and Health Sciences, Grand Forks, North Dakota 58203, USA

**Keywords:** SARS-CoV-2 (Severe Acute Respiratory Syndrome-Coronavirus-2), COVID-19 (Coronavirus Infectious Disease-19), Endolysosomes, ARDS (Acute Respiratory Distress Syndrome), Cytokine Storm

## Abstract

SARS-CoV-2 infection causes COVID-19, which has emerged as a health emergency worldwide. SARS-CoV-2 infects cells by binding to ACE2 receptors and enters into the cytoplasm following its escape from endolysosomes. Once in the cytoplasm, the virus replicates and eventually causes various pathological conditions including acute respiratory distress syndrome (ARDS) that is caused by pro-inflammatory cytokine storms. Thus, endolysosomes and cytokine storms are important therapeutic targets to suppress SARS-CoV-2 infection and COVID-19. Here, we discuss therapeutic targets of SARS-CoV-2 infection and available drugs that could be helpful in the suppression of the SARS-CoV-2 infection and pathological condition COVID-19. The urgency of the COVID-19 pandemic precludes the development of new drugs and increased focus on drug repurposing might provide the quickest way to finding effective medicines.

## Introduction

The high fatality rate and rapidly increasing case numbers of COVID-19 have posed an urgent global health emergency. Contributing to infectivity and confounding containment efforts are large numbers of asymptomatic cases. Currently 208 million people have been infected with SARS-CoV-2 and over 4 million people have died worldwide from COVID-19; infectivity rates and numbers of deaths are particularly high in the USA [1]. This pandemic and health emergency highlights the need for identifying quickly effective therapeutic strategies. However, safe and effective new antiviral drugs usually take more than a decade to develop and therefore drug repurposing might be a better approach.

The SARS-CoV-2 virus has several potential targets against which novel drugs may be developed to suppress viral replication including blocking endocytosis of the virus into cells, viral escape from endolysosomes into the cytoplasm, blocking RNA replication and transcription, inhibiting translation and proteolytic processing of viral proteins, and blocking virion assembly and release from infected cells [2–8]. Suppression of virus-induced cytokine storms can suppress pathological conditions in infected individuals [4,9,10]. Here, we focus attention on the involvement of endolysosomes in SARS-CoV-2 infection and the role that cytokine storms play in the development of COVID-19.

### Endolysosomes

Endosomes and lysosomes (endolysosomes) are acidic organelles [11–13]; a critical feature that is regulated by lysosomes-associated proteins and ion channels including vacuolar-ATPase (v-ATPase) [14–16], two pore channels (TPCs) [17], big-potassium channels (BK) [18], and mucolipin-1 [18]. Endolysosomes play crucial roles in regulating cellular processes like cell cycles and death, metabolism, immune responses and antigen presentation, and membrane trafficking and signaling [19–24]. Endolysosomes have also been implicated in a number of pathological conditions as diverse as cancer, neurological disorders, and viral infections [19,20,24–26]. Different types of viruses use endolysosomes to enter into and infect cells [27–31]. SARS-CoV-2 is endocytosed and then is released into the cytoplasm where it replicates [2,5,32], however much work is still needed to understand better how the virus escapes the endolysosome degradation pathway.

### Cytokine storms

Cytokine storms occur when there is an overproduction of proinflammatory cytokines; a consequence of SARS-CoV-2 infection that disturbs negative feedback regulatory mechanisms of the immune system [33–36]. High levels of pro-inflammatory cytokines are further enhanced because of positive feedback influences on other immune cells which are recruited to sites of inflammation [37,38]. Various cytokines are involved in developing cytokine storms including tumor necrosis factor (TNF), interleukin (IL), colony-stimulating factor (CSF), and interferon (IFN). These virus-induced cytokine storms can lead to the development of ARDS, a systemic inflammatory response that can result in multiple organ failure [38–40]. Thus, cytokine storms are important targets for therapeutic intervention. In Table 1 we list therapeutic drugs that might be studied further for use against COVID-19 and target endolysosomes and cytokine storms.

### Targeting endolysosomes for suppressing SARS-CoV-2 infection

The endolysosome pathway may be a therapeutic target to suppress SARS-CoV-2 infection and COVID-19 [5,41]. The involvement of the endolysosome system starts with SARS-CoV-2 binding to receptors on cell membranes and this is followed by entry into and pH-dependent escape from endolysosomes [5,41]. The spike protein of SARS-CoV-2 is essential for viral entry into cells governed by ACE2 receptor-mediated endocytosis and priming via cellular proteases; all are protected from host immune surveillance [42–44]. Vaccines and antibody-based therapies are challenged by the high binding capacity of the receptors and by ability of spike proteins to escape from host immune surveillance.

Recombinant protein and peptide-based therapies could successfully block virus entry and pathogenic conditions in COVID-19 patients. Indeed, recombinant human ACE2 (APNO1, rhACE2) is currently being developed as a therapeutic target to treat pulmonary arterial hypertension and ARDS [45,46]. Recombinant human ACE2 (rhACE2) protein reduced virus entry into human cell-derived organoids probably acting as a decoy for virus binding [47]. Additional sites for intervention against viral infection include the spike S2 stalk that contains HR1 and HR2 hydrophobic regions; stable six-helix-bundle (6-HB) structures that fuse the virus with host cell membranes. Hence, fusion peptides against HR1 and HR2 hydrophobic regions of spike S2 stalk could be barriers to the virus infection. A lipopeptide derived from EK1, EK1C4, inhibited in nanomolar concentrations SARS-CoV-2 pseudovirus infection and spike protein-mediated membrane fusion [48–50]. Additionally, abelson kinase (ABL) inhibitors (imatinib, dasatinib) blocked cell-fusion of SARS-CoV and MARS-CoV required for virus entry into cells and may similarly protect against SARS-CoV-2 [51].

Following endocytosis, successful viral entry is achieved by proteolytic cleavage of spike proteins catalyzed by the cellular proteases furin, TMPRSS2, and cathepsins; both pH-independently and -dependently [52–59]. Several compounds may block viral infection by restricting endocytosis and proteolytic processing of the spike protein including chlorpromazine, triflupromazine, bufalin & ouabain and camostat mesylate, Z-FY (t-Bu)-DMK, K11777, teicoplanin, MD28170, ONO5335, CQ & HCQ, and lopinavir [59–61].

Chlorpromazine, an anti-schizophrenia drug, inhibits clathrin-mediated endocytosis of the coronaviruses MHV, MERS-CoV, and SARS-CoV [62,63]. Similarly, Na^+^/K^+^-ATPase pump-based inhibitors bufalin and ouabain restricted MERS-CoV infection by inhibiting clathrin-mediated endocytosis [64,65].

Camostat mesylate, an inhibitor of TMPRSS2, is used to treat chronic pancreatitis and it has been shown to suppress SARS-CoV-2 infection in human cells and in mice [59,66]. Clinical trials have begun in Germany and the Netherlands with camostat for COVID-19. Because cathepsins are important for pH-dependent SARS-CoV-2 entry cathepsin L, B, and K inhibitors Z-FY (t-Bu)-DMK, K11777, Teicoplanin, MD28170, and ONO5335 may potentially suppress SARS-CoV-2 infection [67, 68].

CQ and HCQ, anti-malarial drugs that affect endolysosome function, block autophagic flux by deacidifying endolysosomes and inhibit SARS-CoV-2 virus infection in cellular models [69,70]. Both drugs are hyped as prophylaxis drugs against COVID-19; however, their prophylactic effects are not clinically established [71,72]. Both drugs have potential risks of arrhythmia, retinopathy, and reduced antiviral type-1 interferon responses by deactivating RNA sensors (TLR) in endolysosomes [73–78]. Co-administration of IFN-I and HCQ may suppress COVID-19 in patients [79,80] and IFN-I may alleviate HCQ-induced risks by enhancing antiviral responses and autophagy [79,80]. IFN-I-induced autophagy may restrict virus replication by degrading it in lysosomes and potentially enhance antiviral immune responses [34,81].

Endolysosome-associated ion channels (TPCs, NPC1, and v-ATPase) regulate endolysosome pH and thereby affect SARS-CoV-2 infection. TPCs are involved in the entry and trafficking of SARS-CoV-2, MERS-CoV, and Ebola virus [82–84]; the TPC inhibitors tetrandrine, Ned-19 [82], and hanfangchin A significantly inhibited the entry and trafficking of viruses in host cells [68]. Furthermore, apilimod and vaculin-1 restricted SARS-CoV-2 infection by reducing PIKfyve enzyme activity [82,85]; PIKfyve is a regulator of PI (3,5) P2, an endogenous activator of TPCs [86]. Apilimod has antagonistic effects on SARS-CoV-2 infection in primary human lung explants and in human iPSC-derived pneumocyte-like cells [68]. Interestingly, apilimod has also been shown to have a broad-spectrum antiviral activity.

Niemann-Pick disease type C1 (NPC1), an endolysosome-resident protein, is involved in cellular lipid trafficking and the entry of the Ebola virus, MERS-CoV, and SARS-CoV [30,87–90]. NPC1 inhibitors, U1866A and imipramine have broad antiviral activity presumably by deacidifying endolysosomes and accumulating lipids in endolysosomes [91,92]. In addition, cepharanthine, an inhibitor of TPC2 and NPC1, has antiviral activity [93]. Thus, TPCs and NPC1 might attract attention as possible targets to suppress SARS-CoV-2 infection and COVID-19.

v-ATPase is one of the major mechanisms by which pH is regulated in endolysosomes. Endolysosome deacidification by BafA1 inhibits coronavirus infections by targeting the v-ATPase pump [52,59,94]. The SARS-CoV 3CLpro protease de-acidifies endolysosomes by direct interaction with the G1 subunit of v-ATPase and blocks degradation of viral factors [95] thereby enhancing virus replication. Notably, endolysosome acidification may restrict coronavirus infections by blocking the escape of viral RNA to the cytosol, promoting viral degradation in lysosomes, and enhancing autophagy-mediated antiviral responses. Regardless, several compounds acidify endolysosomes and enhance autophagy (Table 1) and might be tested for their ability to suppress SARS-CoV-2 infection.

After SARS-CoV-2 is uncoated and escapes from endolysosomes, the virus is replicated, translated, assembled into new virion particles, and released from infected cells to affect bystander cells. During replication, translated polypeptides are then subjected to autoproteolysis to generate various viral proteins including proteases and RdRp (RNA-dependent RNA polymerase), which could be excellent therapeutic targets because of their crucial roles in virus replication [96]. RdRP plays a vital role in replicating and transcribing viral RNA, making it a suitable and clear target for suppressing virus replication. Several broad-spectrum inhibitors of RdRp including Favipiravir and Remdesivir are either in clinical trials or are approved already for treating infected people [69,97,98]. Both drugs have promising effects against SARS-CoV-2 infection and COVID-19. Additionally, an *in vitro* study using ivermectin, an anti-parasitic drug, showed antiviral effects against SARS-CoV-2; there was reduced mortality rates possibly due to suppression of cytokine storms [99,100].

### Suppressing COVID-19 by targetting cytokine storms

Cytokine storms in COVID-19 patients induces critical pathological conditions by damaging host organs [33,34]. Various treatments may suppress cytokine storms including recombinant ACE2 protein (exogenous RAS modulator) [101], exogenous Ang (1–7) [102], ACE inhibitors and AT1R blockers (irbesartan and losartan) to reduce the proinflammatory effects of Ang II [103], early treatment of type I-interferon (IFN-I) [104], pegylated IFN-lambda [105], and IFN-a2b [106]; protective effects have been observed with lung epithelial cells or upper respiratory tract. Other drugs (melatonin and vitamin D, doxycycline, corticosteroids, anti-TNF-a, IL-6, IL-17, JNK, inhibitors) will be discussed in later sections.

Melatonin has protective effects on vascular endothelial cells and lung tissue by suppressing MMP-9 and IL-6, VEGF, and TNF-α [107,108]. Vitamin D (calcitriol) reduces toll-like receptor-induced cytokine storms; lower plasma levels of vitamin D have been noted in SARS-CoV-2 infected patients and they have a higher risk of hospitalization [109,110]. Vitamin D also attenuated virus-induced cytopathic effects in human respiratory epithelial cells [111]. COVID-19 disease progression is slower in black individuals with high levels of vitamin D, however supplementation with vitamin D did not reduce the severity of COVID-19 compared with placebo [112,113]. Co-administration of vitamin D and melatonin could provide prophylactic protection against COVID-19 [114] because both are inducers of autophagy [115,116].

Doxycycline, a broad-spectrum antibiotic, has protective effects on dengue hemorrhagic fever by suppressing cytokine storms and reducing lymphocyte neutrophils’ infiltration of inflamed tissues [117,118]. Also, doxycycline recovered and reduced disease progression in mild-to-moderate COVID-19 patients with ivermectin treatment [119].

Corticosteroids are generally used to suppress inflammation. However, the duration and timing of these drugs is crucial in the context of COVID-19; early corticosteroid treatment was associated with a high viral load [120]. Steroid administration may be beneficial during cytokine storms and ARDS in COVID-19 patients [121]. Dexamethasone, a corticosteroid, has reduced the mortality rate in COVID-19 patients requiring oxygen with or without invasive ventilation. However, dexamethasone could not reduce mortality risk in patients who did not need respiratory support [122]. The co-administration of tocilizumab and corticosteroids has shown protective effects in non-intubated COVID-19 patients [123]. Estradiol has protective effects in women with SARS-CoV-2 infection by different possible mechanisms [6,124].

Several therapeutic targets are mentioned in Table 1. Therapeutic agents used against cytokine storms include TNF-α inhibitors (Etanercept), IL-6 inhibitors (Tocilizumab, Siltuximab) [125], IL-17 inhibitors (Broadalumab, Sekukinumab) [126], and JNK inhibitors (Fedratinib, Tofacitinib) [127] (Table 1). Etanercept, a TNF-α inhibitor, decreased the risk of developing COVID-19 [128]; thus, it was proposed as a potential first-line choice in SARS-CoV-2 infection based on limited immunogenicity, short half-life, and safety considerations [129]. However, contradictory reports are available with anti-inflammatory drugs related to COVID-19 [130–132].

## Conclusion

The pandemic caused by SARS-CoV-2 infection seriously threatens social-economic development and public health globally even though effective vaccines are becoming increasingly available. However, new variants of SARS-CoV-2 have emerged under selection pressure in different countries; even recently, double mutant strains (like B.1.617) have also emerged. Moreover, mutant strains of SARS-CoV-2 may escape available neutralizing antibodies and pose a new challenge in developing novel therapeutic drugs and vaccines. As suggested, several natural compounds and drugs are currently available for safe use, and randomized, blinded, and controlled clinical trials could test whether these drugs can be repurposed to treat SARS-CoV-2 infection.

## Figures and Tables

**Table 1:** List therapeutic drugs that might be studied further for use against COVID-19 and target endolysosomes and cytokine storms.

Class	Therapeutics Candidates	Potential Mechanism: Mode of Action
Receptor or ligand-based antibody or peptide [49,50] Abelson kinase inhibitors [51]	Vaccine based on coronavirus spike proteins fusion peptides (EK1C4)	Inhibition of virus-host membrane fusion
Cathepsin L inhibitors [52,59,67] Cathepsin K inhibitor [68] Cathepsin D [68] Endocytosis antagonist [62,63] Na+/K+-ATPase inhibitors [64,65] Quinoline [71,72] Adenosine triphosphate analog [98] Pyrazine carboxamide [97] Antihelmintic [99]	Camostat, Z-FY (t-Bu)-DMK, K11777, and Teicoplanin MD28170 and ONO5335 Chloropromazine, triflupromazine Bufalin and Quabain CQ and HCQ Remdesivir Favipiravir Antihelmintic	Inhibition of virus entry Inhibition of virus entry Inhibition of virus entry Inhibition of virus entry Inhibition of virus entry Inhibition of virus entry Reduction of virus replication Reduction of virus replication Reduction of virus replication
Natural hormone supplements [133] Vitamin D [110,111] Steroid hormone [6,120,122] Polyamines [134] Flavone glycoside [135] Stilbenod [7] Beta-hydroxybutyrate, acetone [136] Disaccharide [7] Flavone [137] Flavone [137] Chalconoid [137] Polyphenol [7] Flavanone [7] STA-5326 [68] Bis-benzylisoquinoline [68] Inhibitor of cholesterol trafficking [91] Tricyclic antidepressant (TCA) [92] Anti-fungus [138] Anti-neoplastic compound [93]	Melatonin Calcitriol Estradiol Spermidine and Spermine Baicalin Resveratrol Ketone bodies Trehalose Apigenin Wogonin Butein Curcumin Naringenin Apilimod Hanfangchin A U1866A Imipramine Itraconazole, Posaconazole Cepharanthine	Anti-inflammatory and autophagy inducer Anti-inflammatory and autophagy inducer Anti-inflammatory and autophagy inducer Anti-inflammatory and autophagy inducer Anti-inflammatory and autophagy inducer Anti-inflammatory and autophagy inducer Anti-inflammatory and autophagy inducer Anti-inflammatory and autophagy inducer Anti-inflammatory and autophagy inducer Anti-inflammatory and autophagy inducer Anti-inflammatory and autophagy inducer Anti-inflammatory and autophagy inducer TPCs inhibitor PIKFyve Kinase inhibitor TPCs inhibitor NPC1 inhibitor NPC1 inhibitor NPC1 inhibitor TPCs and NPC1 inhibitor
Exogenous RAS Modulators [45,139] AT1R blocker (ARB) [139] Interferon III [140]	Recombinant ACE2, Ang (1–7), Irbesartan, Iosartan Pegylated INF-λ	Anti-hypertensive Aung (1–7), anti-inflammatory effects Anti-hypertensive Ang (1–7), anti-inflammatory effects Anti-inflammatory and enhance defense of respiration epithelium
Type-1 interferon [141,142] Tetracycline antibiotic [119,143] Glucocorticoid [122] IL-17 inhibitor [144] IL-6 inhibitor [130] TNF inhibitor [128] JAK inhibitor [145]	Interferon Doxycycline Dexamethasone Sekukinumab, Broadalumab Tocilzumab, Siltuximab Etanercept Tofacitinib	Anti-viral and anti-inflammatory Anti-inflammatory response Anti-inflammatory response Anti-inflammatory response Anti-inflammatory response Anti-inflammatory response Anti-inflammatory response
